# Detecting acute reperfusion myocardial hemorrhage with CMR: a translational study

**DOI:** 10.1186/1532-429X-14-S1-O61

**Published:** 2012-02-01

**Authors:** Avinash Kali, Andreas Kumar, Richard L Tang, Rohan Dharmakumar

**Affiliations:** 1 Biomedical Engineering, University of California, Los Angeles, Los Angeles, CA, USA; 2 Biomedical Sciences, Cedars-Sinai Medical Center, Los Angeles, CA, USA; 3 Québec Heart and Lung Institute, Laval University, Québec City, QC, Canada, USA

## Background

Hemorrhage is a frequent hallmark of large acute reperfused myocardial infarctions (rMI). Recently, there has been a growing interest in CMR for noninvasive detection of hemorrhage in rMI. While T2*- and T2-weighted CMR have been used for this purpose, there is a lack of consensus on which of these methods is the most appropriate and reliable. We investigated the relative abilities of T2 and T2* CMR for detecting hemorrhage in rMI in a canine model and patients.

## Methods

Canines (n=14), subjected to ischemia-reperfusion (I/R) injury (3 hrs of LAD occlusion followed by reperfusion), underwent CMR (1.5T) studies on day 5 post reperfusion. T2*-weighted (multi GRE; TE=3.4-18.4ms (6 echoes)), T2-weighted (T2-prep SSFP; prep times=0, 24 and 55 ms), T2-STIR (TE=64 ms) and Late Enhancement (LE) images covering the LV were acquired. Imaging resolution of all the scans was 1.3x1.3x8 mm3.

Patients (n=14) underwent CMR (1.5T) on day 3 post angioplasty for STEMI after providing informed consent. T2*-weighted (TE=2.6-13.8ms (6 echoes)), T2-STIR (TE=61ms) and LE images covering the LV were acquired. Imaging resolution of all the scans was 1.4x1.4x10 mm3.

T2* and T2 maps were constructed by fitting the multi-echo data to monoexponential decay. A threshold-based signal analysis was used to identify hemorrhagic (Hemo+) and non-hemorrhagic (Hemo-) infarcts. T2-STIR signal intensity (STIR-SI), T2* and T2 values, measured from Remote, Hemo- and Hemo+ regions, were compared. Statistical significance was set at p<0.05.

## Results

**Figure 1:**
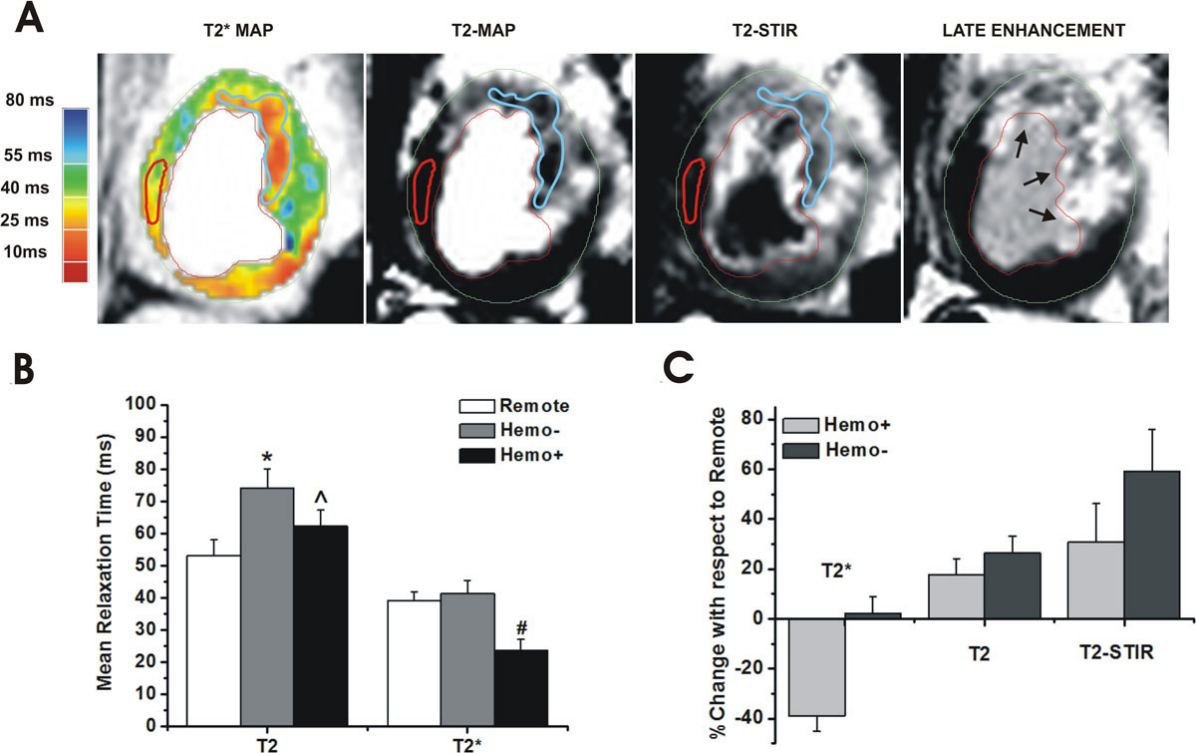
(A) Representative set of T2* (color-coded) and T2 maps, T2-STIR and LE images acquired from a canine on day 5 post reperfusion are shown. Arrows point to the site of infarction on LE image. Hemorrhagic territory (Hemo+) is enclosed in blue ROI, and remote territory is enclosed in red ROI. (B) Mean T2* of Hemo+ was significantly lower than those of Remote and Hemo- (#, p<0.001). Mean T2 of both Hemo+ and Hemo- were elevated compared to Remote (* and ^, p<0.001), with T2 of Hemo- higher than that of Hemo+ (p<0.001). (C) Compared to remote, T2* of Hemo+ decreased by 39% (p<0.001), while T2* of Hemo- remained unchanged (p=0.21). T2 of Hemo- and Hemo+ remained elevated by 26% and 17% respectively (p<0.001 for both cases). STIR-SI of Hemo- and Hemo+ were also elevated by 59% and 31% respectively (p<0.001 for both cases).

Representative T2* and T2 maps, and T2-STIR and LE images (acquired from a canine on day 5 post I/R injury) are shown in Fig. 1A. Mean T2* of Hemo+ was lower than both Remote and Hemo- regions (-39%; p<0.001; Fig. 1B and 1C), while no differences were observed in T2* between Remote and Hemo- (p=0.27). Compared to Remote, mean T2 of both Hemo- and Hemo+ regions were elevated (26% and 17% respectively; p<0.001), with T2 of Hemo- greater than T2 of Hemo+ (p<0.001). A similar trend was observed in T2-STIR images as well; STIR-SI of Hemo+ and Hemo- were greater than Remote (59% and 31% respectively; p<0.001), while STIR-SI of Hemo- was greater than Hemo+ (p<0.001).

Representative T2* map, T2-STIR and LE images, acquired from a patient (day 3 post angioplasty) are shown in Figure 2. Consistent with the animal studies, mean T2* of Hemo+ was lower than the mean T2* of both Remote and Hemo- (-46%, p<0.001; Fig.2), while no differences were observed between T2* of Remote and Hemo- (p=0.61). Mean STIR-SI of both Hemo- and Hemo+ were greater than that of Remote (78% and 33% respectively; p<0.001), with the mean STIR-SI of Hemo- greater than that of Hemo+ (p<0.001).

## Funding

This work was supported in part by grants from American Heart Association (SDG 0735099N) and National Heart, Lung, And Blood Institute (HL091989).

